# How can we design better vaccines to prevent HIV infection in women?

**DOI:** 10.3389/fmicb.2014.00572

**Published:** 2014-11-04

**Authors:** Hannah Rafferty, Sengeziwe Sibeko, Sarah Rowland-Jones

**Affiliations:** Nuffield Department of Medicine, University of OxfordOxford, UK

**Keywords:** vaccines, HIV, women, genital tract, mucosal

## Abstract

The human immunodeficiency virus (HIV) burden in women continues to increase, and heterosexual contact is now the most common route of infection worldwide. Effective protection of women against HIV-1 infection may require a vaccine specifically targeting mucosal immune responses in the female genital tract (FGT). To achieve this goal, a much better understanding of the immunology of the FGT is needed. Here we review the architecture of the immune system of the FGT, recent studies of potential methods to achieve the goal of mucosal protection in women, including systemic-prime, mucosal-boost, FGT-tropic vectors and immune response altering adjuvants. Advances in other fields that enhance our understanding of female genital immune correlates and the interplay between hormonal and immunological systems may also help to achieve protection of women from HIV infection.

## Introduction

The burden of human immunodeficiency virus (HIV) infection in the twenty-first century falls disproportionately on women, particularly in the developing world. Women in Sub-Saharan Africa have, on average, a 60% increased risk of HIV infection compared with their male counterparts (Magadi, 2011) and now account for 58% of HIV-infected adults in the region (UNAIDS, 2012a). Despite these statistics little attention has been paid to developing vaccine candidates that specifically protect the FGT. The holy grail of HIV prophylactics is a vaccine preventing acquisition, achieving “sterilizing immunity.” Unfortunately such vaccines have proven difficult to develop, due to HIV's numerous immune evasion strategies and the speed and strength of immune response required to prevent virus dissemination. Most previous vaccine strategies have focused on achieving systemic immunity with conventional intramuscular immunization. However, HIV is arguably a mucosal disease, with acquisition most common *via* mucosal routes. The ability of HIV to overcome the epithelial barrier and innate immune responses, together with the delayed development of adaptive immune responses, means that there is a very narrow time-window for protection against acquisition at the mucosa. Furthermore, systemic vaccines do not elicit sufficient local immune responses, including secretory Immunoglobulin A (sIgA) (Baral et al., 2012), to prevent infection, so it seems likely that a mucosal strategy such as a vaccine or microbicide, eliciting both systemic and mucosal immune responses, will be most effective in preventing acquisition. Given the biological differences between the immunology of the FGT and other mucosal compartments, a mucosal vaccine specifically targeting the FGT would seem to be the best way to protect women against the spread of HIV.

## Epidemiology of HIV-1 infection in women

Vaginal heterosexual sex is the most common route of transmission worldwide (Kalichman et al., 2011; UNAIDS, 2012b), and women are believed to have double the risk of infection via this route compared to men (Boily et al., 2009). Young women (aged 15–24) are particularly susceptible, accounting for 22% of all new infections (Rodriguez-Garcia et al., 2013). Various factors, both biological and social, may contribute to the high rates of HIV infection in young women. At a social level gender biases are common, particularly in developing countries. The frequency of violence against women combined with their lower socioeconomic status leads to power imbalances. These relationship dynamics give women little ability to negotiate safer sexual practices or the use of contraceptives; hence women are less able to protect themselves actively against infection (Stein, 1990). This is compounded by unequal access to education, with studies suggesting women have consistently poorer knowledge of the benefits of condoms in HIV prevention (UNAIDS, 2012a). In addition, other female populations are pivotal in disease spread. Female sex workers contribute heavily to HIV-1 transmission due to their high HIV prevalence, estimated at 12% worldwide (Baral et al., 2012), along with increased sexual activity. These factors led to direct implication of the sex trade in 10% of Ugandan HIV diagnoses in 2010 (Government of Uganda, 2008). Pregnant women can transmit HIV during pregnancy, labor or breastfeeding, and may also be more likely to acquire HIV than their non-pregnant counterparts (Drake et al., 2014). Effective protection of women is therefore likely to have a large impact on HIV transmission to men and children, especially in high prevalence regions.

## Immunity in the FGT

### Anatomy and immunological structure of the FGT

The FGT can be divided into two distinct regions: the lower consisting of vulva and vagina, and the upper of ovaries, fallopian tubes and uterus, including the ectocervix and endocervix. The vagina was previously thought to be the site of HIV-1 acquisition; however it is now thought that the cervix, particularly the endocervix and the area between the endocervix and ectocervix known as the transformation zone, are particularly susceptible to infection (Nuovo et al., 1993). This is probably due to an abundance of potential HIV target cells, CD4^+^ T-cells, macrophages and dendritic cells, in this region (Pudney et al., 2005), which separates the richly colonized lower reproductive tract and the relatively sterile upper tract. In adolescence, the columnar epithelium of the endocervix extends down into the ectocervix, a phenomenon known as cervical ectopy. This exposes a greater area of more susceptible tissue to potential infection and may contribute to the high risk of HIV infection in adolescent girls.

Cervico-vaginal fluid (CVF) is secreted throughout the FGT mucosa and constitutes the first line of mucosal defense: CVF contains an array of soluble factors including chemokines, cytokines and anti-microbial peptides, many of which have potent anti-HIV activity. Intriguingly, the CVF of younger women, particularly those with cervical ectopy, shows increased levels of pro-inflammatory cytokines (Hwang et al., 2011). This may further increase their susceptibility to HIV infection, as inflammation in the genital tract has been associated with increased HIV infection risk in several studies (Levinson et al., 2009; Naranbhai et al., 2012).

**Figure 1:**
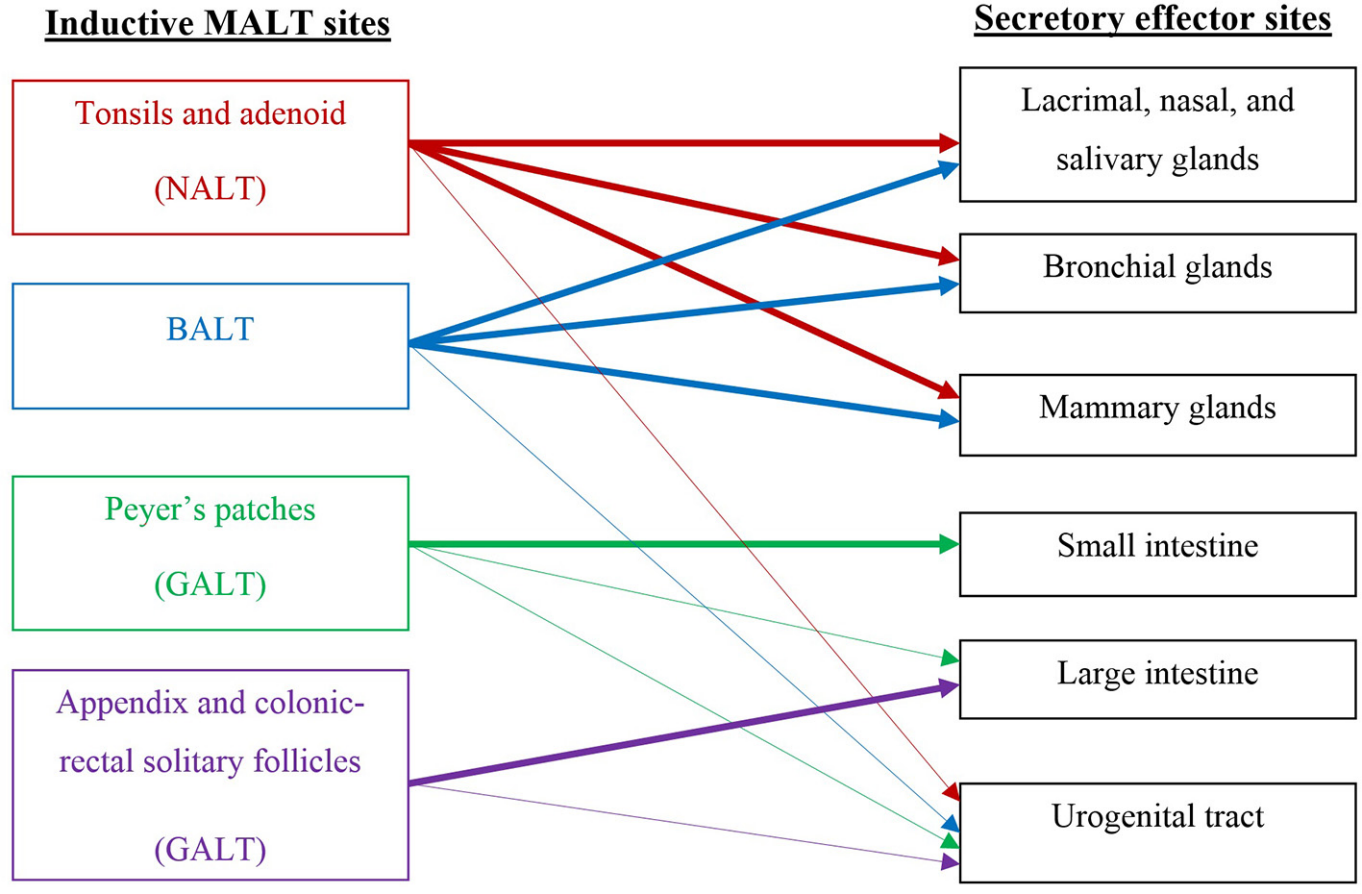
**Schematic of the common mucosal immune system (CMIS) relevant to the stimulation of vaccine-induced responses in the female genital tract**.

The FGT is unique among mucosal surfaces in that it largely lacks organized lymphoid elements, possessing instead small numbers of mononuclear cells scattered throughout the sub-epithelial stroma (Yeaman et al., 1997). This is in marked contrast to the resident immune system of the intestinal mucosa, which consists of clearly-defined lymphoid patches, sub-mucosal lymphocytes, and a large population of intraepithelial lymphocytes poised between crypt epithelial cells (Perry et al., 1998). The absence of a follicular structure means that it is difficult to identify an FGT immune inductive site, responsible for initiating an immune response. Therefore, induction of immunity to genital pathogens is assumed to occur outside the genital tract, followed by recruitment of re-circulating cells into infected sites through the common mucosal immune system (CMIS) (Kantele et al., 1998). There is some evidence that suggests FGT induction sites may be associated with nasal-associated lymphoid tissue (NALT), gut-associated lymphoid tissue (GALT), or bronchial-associated lymphoid tissue (BALT), but none of these preferentially induce local FGT B cells (Mestecky and Russell, 2000) (see Figure 1 for details of general inductive and effector sites). An understanding of the pathways that direct lymphocyte trafficking to the FGT is essential for the development of mucosal vaccines (Perry et al., 1998).

### Mucosal homing

In the mouse, T lymphocyte recruitment to the genital mucosa is directed by the same set of interactions that direct T cells to systemic sites of inflammation, which are distinct from those that dictate traffic to the intestinal mucosa. The homing pathways defined for the intestinal mucosa are assumed to be relevant to all mucosal sites, but are not well represented in the genital tract. This presents yet another area for further investigation if a successful vaccine is to be developed.

## Immunological endocrine interplay

### Hormonal effects on immune responses in the FGT

An important difference between the FGT and other mucosal sites is the influence of female hormones. These not only produce the menstrual cycle, but also affect the immunity of the FGT. Unfortunately this topic has not been extensively researched and hence knowledge relating to vaccine design is limited. It is known that some of the anti-viral proteins in CVF are regulated by hormone status: for example levels of HBD2 and SLP1, two anti-HIV peptides, are lower in CVF during ovulation (Keller et al., 2007). In contrast, oestradiol secretion enhances the secretion of anti-microbial peptides, whilst simultaneously suppressing the secretion of pro-inflammatory cytokines and chemokines (Fahey et al., 2008): these observations would predict lower HIV susceptibility in the first half of the cycle. Macaque studies suggest that females are more susceptible to simian immunodeficiency virus (SIV) vaginal challenge during the luteal (progesterone dominant) phase of the menstrual cycle (Vishwanathan et al., 2011). The luteal phase can be thought of as a time of relative immune suppression in the FGT in order to optimize conditions for fertilization and implantation. The secretion of mucus, as well as anti-microbial peptides, by the endocervix also varies during the menstrual cycle, which may influence susceptibility to infection (Radtke et al., 2012). These factors suggest that there are distinct patterns of immune response and differing susceptibility to infection during the three phases of the menstrual cycle. Although data from human studies are lacking, it has been proposed that women have a distinct “window of vulnerability” to HIV infection in the 7–10 days following ovulation (Wira and Fahey, 2008). Thus future vaccine and microbicide trials need to take account of the menstrual cycle of female participants in order adequately to assess protection from and susceptibility to HIV infection.

## Mucosal transmission

### Initial events in HIV-1 infection in the FGT

Most of our current understanding of the process of HIV mucosal transmission comes from animal models and *in vitro* studies, for example using cervical explants. There is a welcome trend to make SIV models more physiological, with lower doses of SIV used repeatedly in a mucosal challenge. In many previous trials macaques were given high doses of SIV, often intravenously, which is unlikely to represent the early events of HIV-1 infection in women (Haase, 2011). It is still not entirely clear where in the human FGT HIV is most likely to establish primary infection. Transmission studies by Miller and colleagues showed that both vagina and cervix could be sites of primary SIV infection in the SIV/Rhesus macaque model (Miller, 1998). More recent evidence suggests that primary infection takes place predominantly in the cervix following vaginal SIV exposure in macaques, particularly the endocervix and transformation zone (junction between endocervix and ectocervix) (Li et al., 2009a), where the target-cell density [T cells and antigen presenting cells (APCs)] and turnover is greatest (Li et al., 2009b), and where breaks in the mucosa often occur (Norvell et al., 1984).

**Figure 2:**
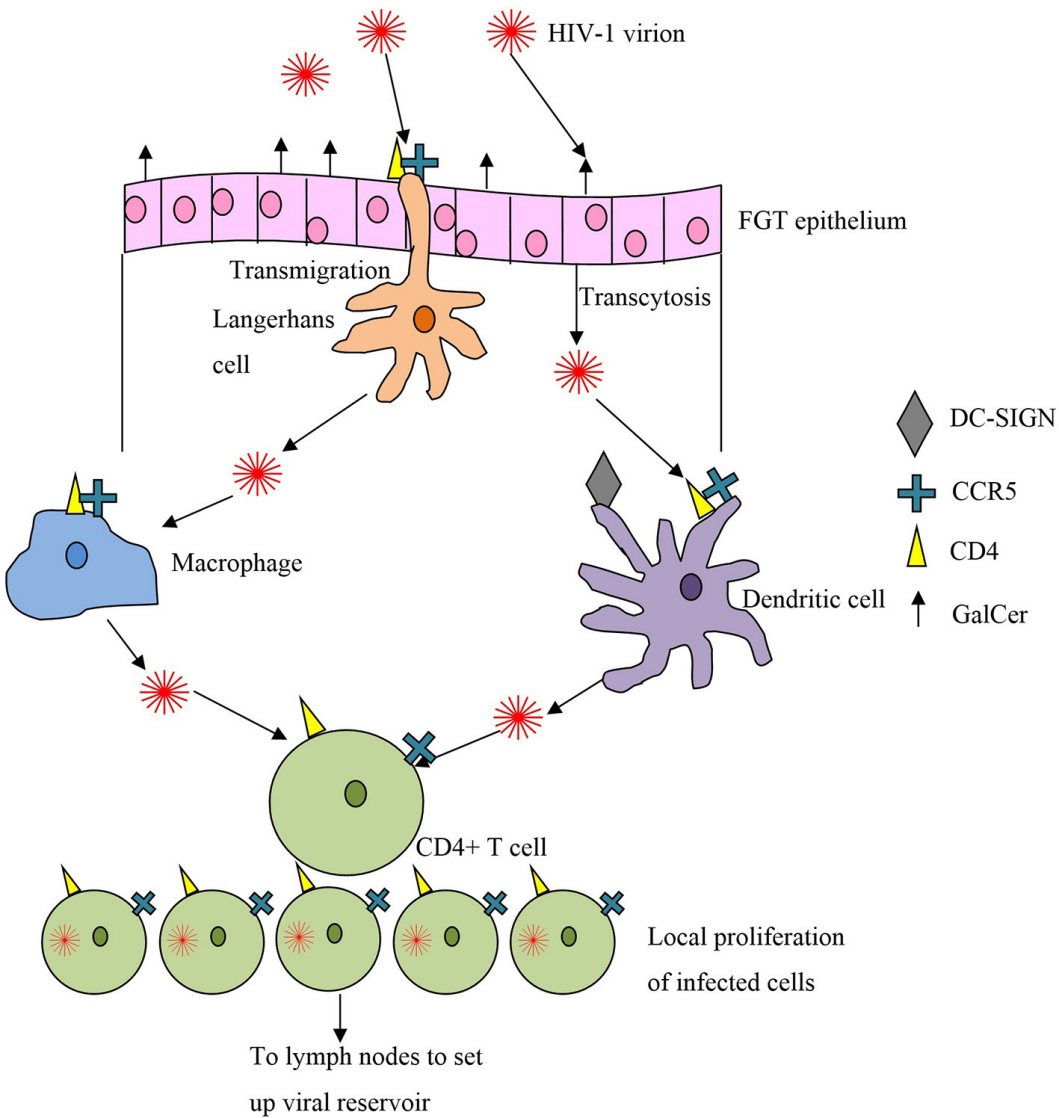
**Crossing the first line of defense, the epithelial mucosa, and targets of HIV infection**.

It was initially thought that HIV first infected vaginal APCs such as macrophages and Langerhans cells, with subsequent rounds of replication occurring in the draining lymph nodes. This was thought to be followed by spread to more proximal lymphoid nodes and finally to the bloodstream and distant lymphoid tissue (Miller, 1998). Subsequently Zhang et al showed that the first cell to be infected is the endocervical intraepithelial resting CD4^+^ T cell (Zhang et al., 1999). Human cervical explant culture models confirmed that memory CD4^+^ T cells were the first infected during HIV transmission across the cervical mucosa (Gupta et al., 2002) (see Figure 2).

Most of our understanding of the very early events following HIV transmission has come from the macaque model of acute SIV infection. Even when large amounts of viral RNA are used in the inoculum, only small foci of tissue-associated viral RNA are found in the first 3–4 days after infection, consistent with a limited founder population of infected cells (Haase, 2011). These clusters of 40–50 infected cells are most consistently found in the endocervix and transformation zone, and expand locally by recruitment of susceptible cells. These observations suggest that there is a critical “window of opportunity” in the first few days after infection, when a targeted immune response involving virus-specific antibodies and/or cytotoxic T lymphocytes (CTL), could control and clear the initial infection before local expansion and subsequent dissemination into the lymphatics. In macaque studies, an influx of SIV-specific CTL was identified that the authors described as generally “too little,” i.e., at too low an effector-to-target ratio to control the infection, and “too late” (Li et al., 2009b).

### Correlates of HIV immunity in the FGT

It is still not clear what responses a vaccine should elicit for protection of the FGT against HIV-1, but some valuable insights have come from studying highly-exposed seronegative subjects (HESNs). SIgA, the major immunoglobulin class involved in mucosal immunity, specific for HIV has been found in the genital fluids of HESN women in several studies (Mazzoli et al., 1997; Devito et al., 2000a,b, 2002; Belec et al., 2001; Broliden et al., 2001; Freeman et al., 2006; Tudor et al., 2009) suggesting it may be important in the protective immune response (Kaul et al., 2001). Further investigations of the HIV IgA response showed these antibodies were directed toward gp41 and were able to inhibit HIV-1 transcytosis and neutralize virions (Devito et al., 2000a,b; Belec et al., 2001; Tudor et al., 2009). HIV-1-specific-immunoglobulin G (IgG) has also been found in the FGT of HESNs (Belec et al., 2001; Buchacz et al., 2001). However, another group found no detectable HIV-1 specific vaginal IgG or IgA in a population of HESNs in the Gambia (Dorrell et al., 2000).

Cellular responses may also contribute to protection against HIV infection. HIV-specific CD8^+^ cytotoxic T-lymphocytes (CTLs) have been detected in the cervical mucosa of HESN sex workers (Kaul et al., 2000, 2003), where they were enriched relative to responses detected in blood (Kaul et al., 2000; Iqbal et al., 2005).

However, others suggest it is not the immune response against HIV-1 that provides protection, but rather the overall immune quiescence of the FGT (Card et al., 2012; Lajoie et al., 2012). This group reported lower levels of pro-inflammatory cytokines in female HESNs compared to HIV-negative controls (Card et al., 2013), as well as a lower level of expression of genes crucial for HIV replication (McLaren et al., 2010; Songok et al., 2012). In contrast, FGT inflammation is associated with an increased risk of HIV infection, presumably due to the recruitment of activated CD4^+^ T-cells (Cohen, 2004; Freeman et al., 2006).

Given that the main function of the FGT is its role in reproduction, it is not surprising that immune tolerance is an important feature of the FGT. Tolerance facilitates fetal implantation in the uterus and allows commensal organisms to colonize the lower tract. Whilst this may contribute to protection against genital infection, tolerance mechanisms, including regulatory T-cells and TGF-β secretion, must therefore be overcome by an induced vaccine response, requiring a highly immunogenic preparation.

## Desirable attributes of a mucosal vaccine

### The need for a mucosal vaccine

**Figure 3:**
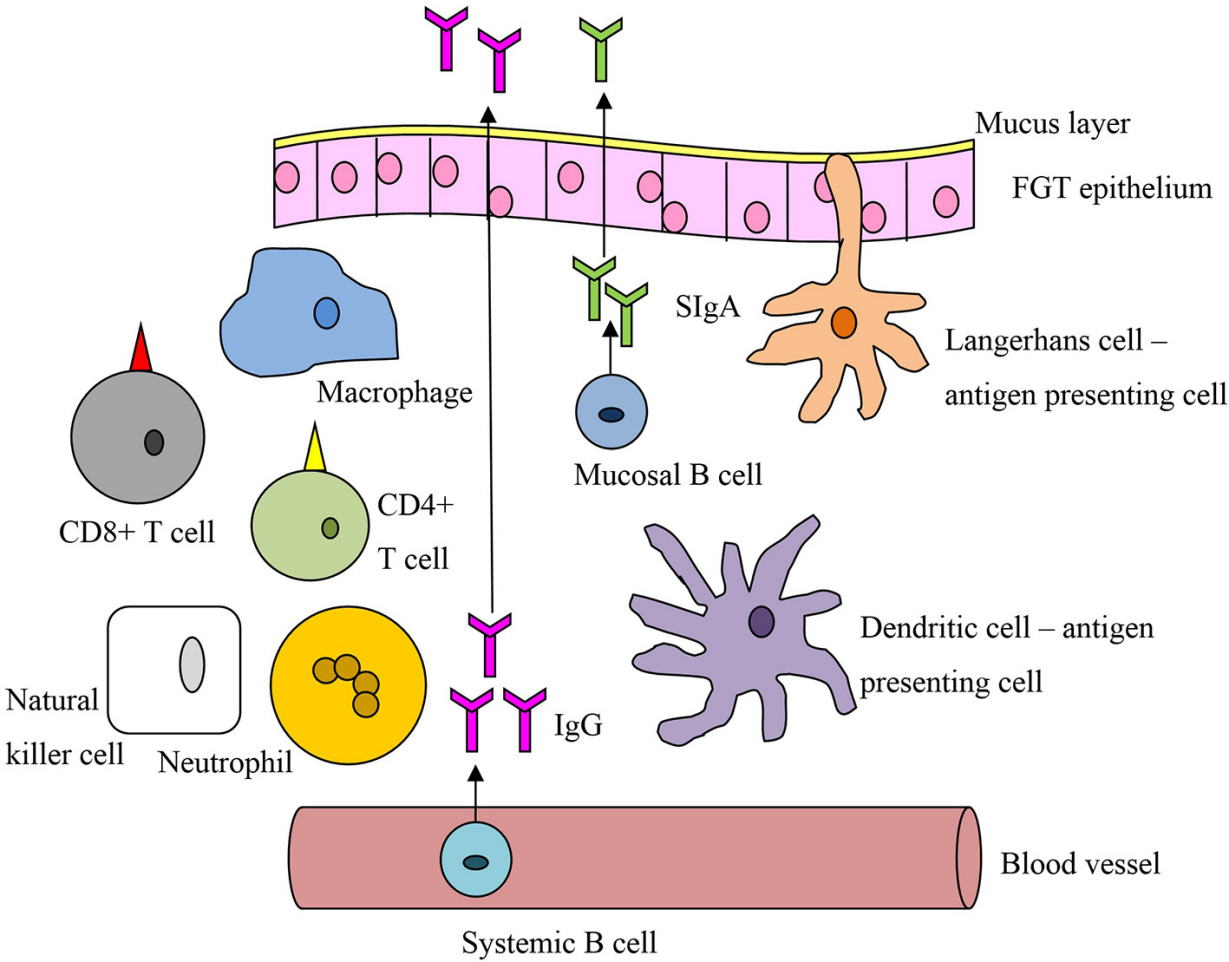
**A general schematic of vaccine-related immune cells active at the mucosal interface of the female genital tract**.

Timing is of the essence to achieve a protective immune response against HIV. The response must be sufficiently rapid to stop the infection before the virus disseminates, by which time it is beyond control (Haase, 2010). Systemic memory responses are too slow to prevent HIV infection at the mucosa; instead a large pool of effector cells at the FGT mucosal surface, ready for immediate mobilization, is more likely to confer protection. Direct comparison of mucosal and systemic vaccination routes has shown the mucosal route alone can induce mucosal memory populations of CTLs (Gallichan and Rosenthal, 1996) and high-avidity CTLs (Ranasinghe and Ramshaw, 2009), matching the CTL profiles of HIV controllers (Mothe et al., 2012). Systemic vaccines can elicit FGT IgG, but in comparison to mucosal vaccines induce little or no SIgA, which is produced locally by plasma cells in the FGT stroma (Nardelli-Haefliger et al., 1999; Pattani et al., 2012). Despite the dominance of the IgG subtype in the lower FGT, SIgA has a decisive role in protection, acting as the first defense against the virus by preventing attachment and hence acquisition (Neutra and Kozlowski, 2006; Brandtzaeg, 2007). Unlike IgG, IgA does not activate the complement system and so can be thought of as anti-inflammatory, important for HIV protection. The impact of a mucosal response to HIV was practically demonstrated by the success of the 1% tenofovir microbicide (Abdool Karim et al., 2010). Mucosal vaccines can also stimulate lymphatic and systemic immune responses (Belyakov et al., 1998), acting as a catch-all for virions that pass into the circulation. These factors suggest a mucosal vaccine specific for the FGT would provide the immune response most likely to prevent HIV acquisition (see Figure 3 and Box 1).

Box 1 **Challenges in developing mucosal vaccines against HIV infection.**

- The gross architecture of the FGT immune system is not typical of other mucosal surfaces—it lacks lymphoid aggregates such as Peyer's patches in the gastrointestinal tract, which comprise B and T cell zones and are responsible for immune induction.
- Correlates of protection against HIV at this site are not well understood for various reasons, including difficulty in establishing appropriate models for studying the HIV-immune system interaction at the mucosal interface.
- Immune tolerance is a feature of the FGT, in view of widespread microbial colonization in its lower tract and the principle function of the upper tract in reproduction—this impedes the mounting of desirable immune responses to locally administered vaccines.
- Systemic immunization, the commonest route of administration of vaccines, is ineffective in generating protective immunity at the mucosa - systemic vaccines do not elicit sufficient local immune responses including SIgA.
- Protein antigens are poor immunogens when given mucosally. Instead of response induction, they lead to immunological tolerance or unresponsiveness known as mucosally-induced tolerance. Consequently, adjuvants are needed to ensure an adequate mucosal immune response is mounted.

### Route of administration and the dosing strategy

As described above, no specific induction site has been characterized in the FGT (see Figure 1), although immune responses can be generated by APCs in the sub-mucosa (Wira and Fahey, 2008). Several possible routes have been investigated including oral, rectal, vaginal, and intranasal. Intranasal administration seems to be a promising strategy in terms of immune response and application. Rhesus macaques showed SIV-specific IgA, IgG and CTLs in cervico-vaginal washes post intranasal immunization with SIV-p55gag with cholera toxin adjuvant (Imaoka et al., 1998). A study in human volunteers also showed an increase in vaginal cholera toxin B (CTB)-specific IgA and IgG with a strong systemic response following intranasal-immunization with CTB subunit (Bergquist et al., 1997). However, a Phase 1 trial of an intranasal HIV-1 vaccine using recombinant HIV-1-gp160 yielded no antibodies in serum or secretory fluids (Pialoux et al., 2008).

It may be better to use a combined prime-boost strategy to elicit FGT immunity. This can, however, be complex as the vaccine response is affected by both the route and the timing of immunization, particularly the interval between the prime and the boost. Rhesus Macaques were immunized with simian-HIV-SF162P3 P1 and recombinant gp41 subunit antigens grafted onto virosomes delivered first intramuscularly (IM) then boosted with intranasal (IN) application (Bomsel et al., 2011). Four of five macaques in the IM/IN group were fully protected against 13 vaginal SHIV challenges delivered over 9 weeks. The other macaques only showed transient infection while none seroconverted to p27gag-SIV. In contrast, only one of six macaques given just the intramuscular formulation was fully protected. All placebo immunized animals seroconverted. Protected animals showed cervicovaginal antigen-specific IgA which inhibited HIV-1 transcytosis and IgG with neutralizing or antibody-dependent cytotoxicity effects. CTLs were not assessed. Some cross-clade transcytosis-inhibition was found in the IM/IN group, suggesting the generation of more broadly neutralizing antibodies. The animals lacked neutralizing antibodies in serum, further emphasizing the importance of a mucosal response for HIV protection.

A recent phase I placebo-controlled trial tested a virosome harboring surface HIV-1 gp41-derived P1 lipidated peptides delivered as an intramuscular prime, then by intranasal boost (Leroux-Roels et al., 2013). The vaccine was safe and well tolerated. P1-specific serum IgG and IgA were induced in all participants receiving the high dose of vaccine. Analysis did not reveal a statistically significant increase in mucosal P1-specifc IgA, despite being detected in 63 and 43% of the low and high dose participants respectively. However, there was an unexpectedly high pre-immune vaginal reactivity toward the P1 antigen, which may have skewed the results. IgA expression is influenced by hormonal changes so sampling during different phases of the menstrual cycle may explain these findings. Vaginal and rectal IgG did increase significantly over the weeks of vaccination for both the high and the low dose groups. These vaginal antibodies were further investigated and were shown to possess the ability to inhibit HIV-1 transcytosis. This result shows promise, demonstrating both the safety and immunogenicity of mucosal vaccines.

A study looking at HSV-2 infection used a novel prime-boost strategy known as “prime-pull,” which could be extrapolated to an HIV vaccine (Shin and Iwasaki, 2012). The “prime-pull” technique involves the mucosal application of chemokines after immunization to recruit primed cells to the mucosa. Mice were immunized systemically, then chemokines CXCL9 and CXCL10 were applied directly to the vaginal mucosa. CTLs were recruited to the FGT and established a long-term population of memory CD8^+^ T cells. CD4^+^ T cells were initially recruited to the FGT but not retained there long-term. The initial CD4^+^ T-cell influx could potentially increase HIV risk, but after the effector phase these cells withdrew, so overall the risk is deemed negligible. In addition, no markers of inflammation were found, and the strategy led to complete protection from vaginal HSV-2 infection.

Following this promising result the “prime-pull” technique was recently adapted to HIV. Mice were immunized intranasally with HIV-1-gp140 and then either the cytokine CCL28 or the toll-like receptor 4 ligand (TLR4) MPLA was administered to the vaginal mucosa (Tregoning et al., 2013). The application of CCL28 post-intranasal vaccine did not increase vaginal B cells or antibodies; however MPLA application caused significant increases in HIV-1 specific vaginal IgA and serum IgG and IgA. The authors concede that, as homing mechanisms in the urogenital tract are not well understood, CCL28 may not have been the best cytokine to use, and suggest that other chemokines involved in B-cell recruitment should be tested. It may be that a single cytokine is insufficient and several acting in concert would provide a better “pull” toward the urogenital tract. On the other hand the success of MPLA adds another dimension to this “prime-pull” strategy; different TLR agonists should also now be investigated.

### Adjuvants

As seen with the “prime-pull” strategy described above, most vaccines require an adjuvant to boost the immunogenicity of the construct. Adjuvants are substances that possess the biological capacity to enhance, prolong or accelerate the quality of specific immune responses to vaccine antigens. With regards to mucosal vaccines, adjuvants can be broadly classified into those that play an immunostimulatory role and those that facilitate vaccine delivery for the induction of protective immunity via the CMIS (Yuki and Kiyono, 2003).

Few adjuvant studies have focused on boosting mucosal HIV-specific immunity. Chemokines and cytokines are widely thought to be the most effective adjuvants for HIV-1 vaccines. When CCL28 was used to adjuvant an HIV-1_IIIB_ virus like particle (VLP) construct, enhanced neutralizing capabilities against HIV-1 clade B laboratory isolates and an HIV-1 clade C primary isolate were found in vaginal secretions and sera of mice (Rainone et al., 2011). Increased env-specific interferon gamma (IFN-γ) and interleukin (IL)-45 were also seen, with increased serum IgA, both non-specific and specific for HIV-1. More recently, mice were immunized either IM or IN with HIV-1 gp140 co-delivered with plasmid CCL19 or CCL28 (Hu et al., 2013). Both IM and IN protocols enhanced serum IgG responses, and both cytokines enhanced vaginal IgG and IgA responses, but only when given via the IN route. The vaginal antibodies showed neutralizing activity against both homologous and heterologous HIV-1.

A novel approach to using cytokines as adjuvants employed soluble IL-13 receptors to antagonize the IL-13 response (Ranasinghe et al., 2013). Recombinant poxviruses that co-expressed HIV-1-gag/pol with IL-13Rα2 soluble receptors were given via intranasal-prime, intramuscular-boost to female mice. The transient blockade of IL-13 resulted in the generation of high-avidity CTLs in the iliac and genito-rectal nodes (which drain the FGT), which were not found in the control protocol without soluble IL-13Rα2: high-avidity CTLs were more protective, shown by greater protection following an intranasal challenge with gag-expressing influenza in the IL-13Rα2 group. The stimulation of high-avidity CTLs matches the CTL profiles of HIV controllers (Mothe et al., 2012). This study presents an interesting alternative to the conventional addition of cytokines. Potentially a mixture of addition and blockade of cytokine pathways will generate a suitable immune response in the FGT.

## FGT tropic vectors

Given the mucosal site of HIV acquisition, effective vectors should exhibit mucosal tropism, ideally specific for the FGT. Adenovirus, a commonly used vector, targets the mucosa but is not FGT-specific: this may contribute to its lack of success in clinical trials to prevent HIV infection. There is controversy surrounding the use of adenovirus as a vector after the STEP trial (HVTN502/Merck023), the first clinical trial to examine an HIV prophylactic vaccine using adenovirus as a vector, suggested that adenovirus priming may actually increase the risk of HIV infection (Buchbinder et al., 2008).

Human papillomavirus (HPV) is a FGT tropic virus that infects cervicovaginal keratinocytes, lying senescent for long periods of time. A recent study in Cynomolgus and Rhesus Macaques used HPV pseudovirosomes to deliver SIV-Gag-DNA (Gordon et al., 2012). Gag-specific IgA, IgG and CD4/8+ T cell responses were found in the serum and vaginal tract, which rapidly expanded following intravaginal SIV exposure, suggesting the formation of memory populations. However limitations of this study were that only low levels of vaginal IgG and IgA were induced, and the vaccine led to a substantial CD4^+^ T cell response that could increase HIV susceptibility: there was no protection against vaginal SIV challenge. Furthermore, unattenuated HPV has been shown to increase CXCL8 levels, potentially increasing susceptibility to HIV-1 in cervical tissues and upregulating HIV-1 proliferation (Narimatsu et al., 2005).

These are early days in mucosal targeting of vaccines: with subsequent testing and refining, better results may be achieved. Other sexually transmitted disease (STD) vectors such as HSV-2 could also be tested as vectors. HSV-1 has been used as a vector in mice (Parker et al., 2007), using an HSV-1 vector expressing the HIV-1 gag gene for intraperitoneal immunization. Strong gag-specific CD8+ responses were elicited (Parker et al., 2007) which persisted 9 months post-immunization. HSV-2 is closely related to HSV-1, but is acquired through the genital mucosa suggesting it may be a more appropriate HIV vaccine vector. However, further research is needed to improve immune responses and reduce potentially harmful mucosal inflammation.

## Future directions

Despite the possibilities highlighted in these studies, several factors must be addressed to improve the development of an effective HIV vaccine. We strongly recommend that immune responses in the FGT should be measured as an integral part of every HIV vaccine trial. Even though precise correlates of immunity are not yet known, it seems reasonable to assume that local immune responses to HIV in the FGT will play an important part in protection against sexual acquisition. Ensuring that FGT responses are always measured provides a timely reminder of the site where most HIV infections in the world are acquired, and should inform future trials and vaccine design. Correlates of FGT inflammation should also be investigated to ensure a vaccine does not increase HIV susceptibility. A focus on FGT responses requires a distinct agenda from the outset so that a laboratory science program is incorporated and prioritized within the parent clinical trial protocol. It is also important to study participants with breakthrough infections, and collect and store relevant, appropriate, and appropriately timed biological specimens, collected as close as possible to the estimated time of infection, (Sibeko and Makvandi-Nejad, 2013).

Our lack of knowledge in key aspects of FGT immunology remains a major problem for mucosal vaccine development. Characterization of FGT homing pathways would greatly improve mucosal vaccine design, as would clear evidence of which compartment in the human FGT is most susceptible to HIV infection. HIV predominantly affects women in developing countries, and therefore for maximal public health benefit new vaccines should be cheap, store well, and preferably not require administration by medical practitioners.

Despite having a significant impact on the immunology of the FGT, the changing levels of estrogen and progesterone throughout the menstrual cycle have rarely been considered in natural history or vaccine studies (Hickey et al., 2011). A better understanding of the immunological milieu in different menstrual phases may suggest a specific point of the cycle when vaccination would be most beneficial. Previous studies suggest that intravaginal vaccination is most effective in women during the follicular phase of the cycle (Kozlowski et al., 2002). The effects of hormones on other mucosal routes have not yet been characterized. The cycle stage should therefore be an important consideration in vaccine trials, and vaccines should be tested during different hormonal stages to assess the most effective timing of administration. Adolescence is an important period when major hormonal fluctuations occur. The HIV-1 incidence in the 15–24 year age group is twice as high in women compared to men (UNAIDS, 2012a), highlighting the importance and challenges of vaccine efficacy in this vulnerable group.

Differential immune responses for adults and adolescents against HSV-2 and *E. Coli* suggest that correlates of mucosal immunity may differ substantially in different age-groups (Madan et al., 2012). It may therefore be necessary to look systematically at different populations and to include adolescents in future vaccine trials. Furthermore, substantial hormonal fluctuations also occur during pregnancy and with the use of hormonal contraceptives. In the future, vaccine assessment should extend to these groups in clinical trials to ensure optimal protection of women at different stages of life.

## Conclusions

The dynamics of HIV infection are changing, with more women infected than in previous years and at younger ages than their male counterparts. This trend probably reflects a combination of socioeconomic and biological factors. Preventing infection in women will have a major impact on HIV incidence in their partners and children. A vaccine specifically targeting the FGT may be needed to induce an immune response that is able to contain HIV prior to dissemination. As mucosal vaccines induce SIgA and mucosal CTL memory responses more successfully than their systemic counterparts, a mucosal vaccine specifically targeting the FGT appears to be the best option for preventing HIV acquisition. Several strategies look promising, with systemic-prime, mucosal-boost, FGT tropic vectors, and adjuvants tailoring the immune response to the FGT all yielding encouraging results in animal models. However, much more must be done: future vaccine trials must put more emphasis on the immune responses of the FGT and consider hormonal effects on mucosal immunity from the outset. Further research on mucosal vaccines specifically targeting these issues may finally yield the protective vaccine needed to protect women and the wider population from the spread of HIV.

### Conflict of interest statement

The authors declare that the research was conducted in the absence of any commercial or financial relationships that could be construed as a potential conflict of interest.
